# Retraction: Tanshinone IIA inhibits HIF-1α and VEGF expression in breast cancer cells via mTOR/p70S6K/RPS6/4E-BP1 signaling pathway

**DOI:** 10.1371/journal.pone.0321151

**Published:** 2025-03-21

**Authors:** The PLOS One Editors

After this article [1] was published, the corresponding author contacted PLOS requesting retraction of [1] due to concerns about the tumor volumes reported in Figs 5A and 5C, which exceed internationally-accepted animal welfare standards. The corresponding author noted that at the time of publication of [1] there were no regulations in China on ethical tumor volume endpoints in mice.

During editorial reassessment of the article additional concerns were raised regarding results presented in Figs 1, 3, and 4. Specifically:

- In multiple panels, two or more lanes within a single western blot panel appear similar to each other, including:
  - In Fig 1A, within the NE HIF-1β panel and within the WCE HIF-1α panel.
  - In Fig 1B, within the NE HIF-1β panel and within the WCE HIF-2α panel.
  - In Fig 3B, within the [^35^S]HIF-1α Normoxia panel.
  - In Fig 3C, within the [^35^S]HIF-1α Normoxia panel.
  - In Fig 4A, within the mTOR, p-p70S6K(Thr421/Ser424), p-p70S6K(Thr389), p70S6K, p-4E-BP1 (Thr37/46), and 4E-BP1 panels.
  - In Fig 4B, within the p-mTOR, p-p70S6K, and β-actin panels.
- The Fig 1A NE HIF-1α panel appears similar to the Fig 1B NE HIF-1α panel, despite representing different experimental conditions.
- Fig 1B WCE HIF-1α lanes 8-10 appear similar to Fig 3B [^35^S]HIF-1α Normoxia lanes 4–6.
- There appear to be multiple vertical discontinuities in the panels presented in Fig 4B.

The corresponding author stated that an error was made in the preparation of Fig 1B NE HIF-1α panel, and they provided a replacement image for this panel. However, the corresponding author disagreed with the additional concerns raised with Figs 1, 3 and 4 listed above, and stated there was no splicing of the above-listed western blot panels. They indicated that the majority of raw blot images underlying the published panels listed above are no longer available. PLOS remains concerned that areas in the Figs 1, 3 and 4 panels listed above appear more similar than would be expected from independent results. In the absence of the underlying data, the concerns cannot be resolved.

In light of the above unresolved concerns, the *PLOS One* Editors retract this article.

NG agreed with the retraction. GL, CS, LL, TZ, JZ, XH, YC, and HC either did not respond directly or could not be reached.
