# Correction: Retraction: Tanshinone IIA Inhibits HIF-1α and VEGF Expression in Breast Cancer Cells via mTOR/p70S6K/RPS6/4E-BP1 Signaling Pathway

**DOI:** 10.1371/journal.pone.0324274

**Published:** 2025-05-12

**Authors:** The PLOS One Editors

## Notice of republication

The retraction notice [1] for this article [2] was republished on April 22, 2025, to correct an error in the author agreement statement. The publisher apologizes for the error.

In addition, at the time of republication the wording of the retraction notice was amended to clarify that the corresponding author contacted PLOS requesting retraction of their article following tumor volume concerns, and to clarify that the corresponding author confirms the Fig 1B NE HIF-1α panel is incorrect, but disagrees with the additional image concerns observed by PLOS during editorial reassessment.

Please download this retraction notice again to view the correct version. The originally published, uncorrected notice and the republished, corrected notice are provided here for reference.

## Supporting information

S1 File Originally published, uncorrected retraction notice. (PDF)

S2 File Republished, corrected retraction notice. (PDF)
