# Supplementary material for: Correction: Retraction: Tanshinone IIA Inhibits HIF-1α and VEGF Expression in Breast Cancer Cells via mTOR/p70S6K/RPS6/4E-BP1 Signaling Pathway
Source: PLoS One. 2025 May 12;20(5):e0324274. doi: 10.1371/journal.pone.0324274 (PMC12068618; doi:10.1371/journal.pone.0324274)
Supplement: S2 File — (PDF) [file pone.0324274.s002.pdf]

PLOS ONE

RETRACTION

Retraction: Tanshinone IIA inhibits HIF-1α and VEGF expression in breast cancer cells via mTOR/p70S6K/RPS6/4E-BP1 signaling pathway

The *PLOS One* Editors

After this article [1] was published, the corresponding author contacted PLOS requesting retraction of [1] due to concerns about the tumor volumes reported in Figs 5A and 5C, which exceed internationally-accepted animal welfare standards. The corresponding author noted that at the time of publication of [1] there were no regulations in China on ethical tumor volume endpoints in mice.

During editorial reassessment of the article additional concerns were raised regarding results presented in Figs 1, 3, and 4. Specifically:

- In multiple panels, two or more lanes within a single western blot panel appear similar to each other, including:
  - In Fig 1A, within the NE HIF-1β panel and within the WCE HIF-1α panel.
  - In Fig 1B, within the NE HIF-1β panel and within the WCE HIF-2α panel.
  - In Fig 3B, within the [^{35}S]HIF-1α Normoxia panel.
  - In Fig 3C, within the [^{35}S]HIF-1α Normoxia panel.
  - In Fig 4A, within the mTOR, p-p70S6K(Thr421/Ser424), p-p70S6K(Thr389), p70S6K, p-4E-BP1 (Thr37/46), and 4E-BP1 panels.
  - In Fig 4B, within the p-mTOR, p-p70S6K, and β-actin panels.
- The Fig 1A NE HIF-1α panel appears similar to the Fig 1B NE HIF-1α panel, despite representing different experimental conditions.
- Fig 1B WCE HIF-1α lanes 8-10 appear similar to Fig 3B [^{35}S]HIF-1α Normoxia lanes 4–6.
- There appear to be multiple vertical discontinuities in the panels presented in Fig 4B.

The corresponding author stated that an error was made in the preparation of Fig 1B NE HIF-1α panel, and they provided a replacement image for this panel. However, the corresponding author disagreed with the additional concerns raised with Figs 1, 3 and 4 listed above, and stated there was no splicing of the above-listed western blot panels. They indicated that the majority of raw blot images underlying the published panels listed above are no longer available. PLOS remains concerned that areas in the Figs 1, 3 and 4 panels listed above appear more similar than would be expected from independent results. In the absence of the underlying data, the concerns cannot be resolved.

In light of the above unresolved concerns, the *PLOS One* Editors retract this article.

NG agreed with the retraction. GL, CS, LL, TZ, JZ, XH, YC, and HC either did not respond directly or could not be reached.

Check for updates

OPEN ACCESS

Citation: The *PLOS One* Editors (2025) Retraction: Tanshinone IIA Inhibits HIF-1α and VEGF Expression in Breast Cancer Cells via mTOR/p70S6K/RPS6/4E-BP1 Signaling Pathway. PLoS ONE 20(3): e0321151. https://doi.org/10.1371/journal.pone.0321151

Published: March 21, 2025

Copyright: © 2025 The *PLOS One* Editors. This is an open access article distributed under the terms of the Creative Commons Attribution License, which permits unrestricted use, distribution, and reproduction in any medium, provided the original author and source are credited.

Reference

1. Li G, Shan C, Liu L, Zhou T, Zhou J, Hu X, et al. Tanshinone IIA Inhibits HIF-1α and VEGF Expression in Breast Cancer Cells via mTOR/p70S6K/RPS6/4E-BP1 Signaling Pathway. PLoS ONE. 2015;10(2):e0117440. https://doi.org/10.1371/journal.pone.0117440
